# A Unique Case of Deficiency of Adenosine Deaminase 2 Single in a Young Adult Patient

**DOI:** 10.7759/cureus.33273

**Published:** 2023-01-02

**Authors:** Atheer Alharthi, Ghaith R Alhashmi Alamer, Saif S Alqurashi, Emad E Alsaeedi, Hasheema Alsulami

**Affiliations:** 1 Medicine and Surgery, Umm Al-Qura University, Makkah, SAU; 2 College of Medicine, Umm Al-Qura University, Makkah, SAU; 3 Internal Medicine and Rheumatology, King Abdullah Medical City, Makkah, SAU

**Keywords:** adult onset, saudi arabia., case report, deficiency of adenosine deaminase 2, dada2

## Abstract

Deficiency of adenosine deaminase 2 (DADA2) is a rare monogenic disease caused by mutations in the adenosine deaminase 2 gene (ADA2). It is characterized by a wide range of manifestations, including systemic inflammation and vasculopathy, early onset stroke (ischemic or hemorrhagic), immunodeficiency, and bone marrow failure. The diagnosis of DADA2 is confirmed by pathogenic mutations in ADA2 or low ADA2 enzymatic activity in the patient. In this study, we present a case of a 24-year-old Saudi male who was admitted with symptomatic anemia, lightheadedness, exertional symptoms, and a history of fever (38.1 C) for one week. Laboratory tests revealed normocytic normochromic anemia, leucopenia, lymphopenia, and neutropenia-autoimmune profile: low C3 and positive anti-ds DNA. The genetic testing revealed two Pathogenic variants, which were identified in ADA2. The diagnosis of DADA2 was made, and the patient received subcutaneous adalimumab 40 mg every two weeks. At the follow-up after one month, he showed improvement in fever, rash, and C-reactive protein (CRP) from (6 to 0.65).

In conclusion, we present one of the first cases in Saudi Arabia of an adult patient diagnosed with DADA2 with a unique gene mutation. Adult-onset patients with DADA2 usually have a vague presentation and a relatively narrower phenotype range of symptoms which produce additional challenges for the physician to add DADA2 to the list of differentials. We suggest further studies investigate the genotype-phenotype association, possible clinical presentation, and the development of curative treatments for those cases.

## Introduction

Deficiency of adenosine deaminase 2 (DADA2) is a rare monogenic disease caused by DNA mutations in the adenosine deaminase 2 gene (ADA2). Formerly known as cat eye syndrome chromosome region, candidate1 (CECR1), which is located on chromosome 22q11, and is characterized by systemic inflammation and vasculopathy that impacts a wide variety of organs. DADA2 was identified as a type of systemic vasculitis that resembled polyarteritis nodosa [1,2].

Manifestations of the disease include but are not limited to, early-onset stroke (ischemic and hemorrhagic), immunodeficiency, bone marrow failure, recurrent fever, livedoid rash (reticularis or racemosa), and various cytopenias. Such wide-spectrum clinical manifestations require assessment by many sub-specialties, increasing the difficulty of diagnosis and early detection of the disease [3,4].

Early diagnosis and management are essential, given the morbidity and mortality rate [4]. Confirmation of known pathogenic mutations in ADA2 or low ADA2 enzymatic activity in the patient is diagnostic for DADA2. Screening, which is crucial to detect the disease early, is yet to have clear indications. However, when encountering inflammation with vasculitis, screening may be considered. Early treatment with tumor necrosis factor (TNF) inhibitors soon after diagnosis results in significant improvement of the disease [5,6].

Since the discovery of DADA2 in 2014, about 260 instances of the disease have been documented as of 2020 [6]. The prevalence of the disease might be as high as four per 100,000 people, and it is expected that both men and women are affected equally, given its autosomal recessive nature of inheritance. DADA2 is more prevalent generally in groups that have high levels of consanguinity [7].

## Case presentation

A 24-year-old Saudi male was admitted to the emergency department with symptomatic anemia palpitation, lightheadedness, exertional symptoms, and a history of fever for one week. Initially intermittent, then became a continuous pattern of symptoms the day prior to presentation occurred. There was no history of headache, weakness, hearing loss, or vision loss. His past medical history included recurrent severe symptomatic anemia requiring blood transfusion almost weekly and unexplained subjective fever for the four months prior to presentation. He had been diagnosed with Raynaud's phenomenon a year before presentation, after experiencing pain in his lower limbs and skin discoloration, and was started on aspirin (ASA) with no improvement. He subsequently stopped ASA of his own volition. He had a recurrent herpes zoster infection, with three episodes in total, the last one being three months ago. He had been treated with valacyclovir one gm three times a day for 10 days, and while healing occurred, this was complicated by neuropathic pain. He additionally had a history of multiple joint pain without morning stiffness.

The patient had been healthy, with no known disorders in childhood, and had been fully vaccinated without complications. The patient was born to healthy parents who were cousins. Reviewing the family profile, the patient's older sister complained of severe and recurrent episodes of anemia, mouth ulcers, and abscesses. She was diagnosed with Bechet's disease and died at the age of 27 years.

The patient was febrile (38.1°C) on physical examination with stable vital signs-bilateral cervical lymphadenopathy. On abdominal examination, we observed hepatosplenomegaly. Skin eruption was noted over the left chest wall and back, and there was lower limb edema with skin hypopigmentation in both limbs. Laboratory tests showed normocytic normochromic anemia (hemoglobin (HB) 5.6 gm/dl, mean corpuscular volume (MCV) 82.0 FL, mean corpuscular hemoglobin (MCH) 27.3 pg), leucopenia (white blood cells (WBCs) 3.60*109/L), lymphopenia (lymphocytes 0.22*109/L), and neutropenia (neutrophil 2.13*109/L) (Table 1). Viral serology was negative, hemolytic workup direct antiglobulin test (DAT) was negative, bilirubin levels were 0.4, lactate dehydrogenase (LDH) was 155, and haptoglobin was 222. His autoimmune profile was as follows: anti-neutrophil cytoplasm antibodies was negative, C3 was low, C4 was normal, anti-double-standed was DNA positive, antinuclear antibodies (ANA) were negative, and thyroid profile was normal.

**Table 1:** Laboratory data HGB - hemoglobin, MCV - mean corpuscular volume, MCH - mean corpuscular hemoglobin, WBC - white blood cell

Test item	Values	Normal values
HGB	5.6 gm/dl	13.5-17.5
MCV	83.8 fL	80-100
MCH	28.4 pg	27-32
WBCs	2.44*109/L	3.9-11
Lactate dehydrogenase (LD) serum	236.00 U/L	87-241
Bilirubin total	0.4 mg/dL	<1.2
C3	74.8 mg/dL	79.0-152.0
C4	16.0 mg/dL	16.0-38.0
Anti Scl-70 (topoisomerase -1)	0.05 (ratio)	Negative <1
Antinuclear antibodies (ANA)	0.23 (index)	Negative <1
ANCA (c-ANCA & p-ANCA)	Anti - PR3: 0.39 (index) Anti - MPO: 0.52 (index)	Negative <1
Anti-Beta-2 glycoprotein 1 Abs IgG	2.4 U/mL	Negative <7
Anti-Beta-2 Glycoprotein 1 Abs IgM	1.3 U/mL	Negative <7
Anti - Sm	0.04 (Ratio)	Negative <1
Anti - Cardiolipin Abs	Anti-cardiolipin IgG: 26.40 U/mL; anti-cardiolipin IgM: 25.9 U/mL	<48.00; <44
Anti -dsDNA , Abs	193 IU/mL	0-30
Rheumatoid Factor	59.2 IU/mL	Negative <20
Haptoglobin	222 mg/dL	36-195
Thyroid stimulating hormone	4.176 mIU/L	0.55-4.78
Flowcytometric quantitation of T, B, and NK lymphocytes		
T Cell (CD3)	91%	59-83
T Cell (CD3), absolute	182 cells/mcL	677-2383
Helper T cells (CD4)	66%	31-59
Helper T cells (CD4), absolute	132 cells/mcL	424-1509
Suppressor T cells (CD8)	21%	12-38
Suppressor T cells (CD8), absolute	42 cells/mcL	169-955
H/S (CD4+/CD8+) ratio	3.2%	= or >1
B cells (CD19)	1%	6-22
B cells (CD19), absolute	2 cells/mcL	99-527
Natural killer cells (CD16+CD56)	7%	6-27
Natural killer cells (CD16+CD56), absolute	14 cells/mcL	101-678

Genetic testing revealed two pathogenic variants (c.882-2A>G splice acceptor (homozygous)) identified in ADA2, which is associated with autosomal recessive DADA2. Magnetic resonance imaging of the brain was performed to rule out stroke, as this is a risk for these patients. Given the patient's hepatomegaly and lower limb edema, a doppler ultrasound of the liver was done to rule out portal hypertension. Lymph node biopsy was also performed to rule out lymphoma, as patients with immune disorders can be at risk for this disease.

A diagnosis of DADA2 was made, and the patient received subcutaneous adalimumab 40 mg every two weeks. In addition, given his recurrent varicella-zoster virus, he was started on prophylactic acyclovir 800 mg two times a day to monitor renal function. At the follow-up after one month, he showed improvement in fever, rash, and C-reactive protein (from 6 to 0.65). However, his anemia remained almost the same; he is blood transfusion dependent, and has recently been started on chelation therapy.

## Discussion

In the current study, we present the first case in Saudi Arabia of an adult patient diagnosed with DADA2 with a unique gene mutation. It is essential to have insight into the variation of phenotypes and genotypes of DADA2, as clinical manifestations may differ according to onset age. A previous study by Zhang et al. [8] revealed that lower frequencies in all three symptoms (vasculopathy, systemic inflammation, and hematological abnormalities) were noticed among adult-onset patients compared with pediatric patients, who usually have more frequent symptoms. Sharma et al. conducted a case series study that revealed similar results. They reported lower frequencies of all three symptoms among adults and found the most common manifestations among adult patients to be hematological abnormalities [9]. The narrow phenotype range of the disease among adult patients may add additional detection and diagnostic challenges for physicians. Our patient presented with a picture of anemia in addition to fever only, and this vague presentation sequentially lengthened the list of differential diagnoses. Several cases worldwide have reported vague presentations similar to our patient [10,11]. Although numerous studies in the literature report on neurological involvement, an early-onset and recurrent history of strokes is a hallmark clinical feature of DADA2 (prevalence of 42%) [1].

This condition is autosomal recessive inheritance, which requires both parents to be gene carriers for the offspring to be affected. [7] Such is our case; both parents were cousins. Timely gene sequencing and measurements of ADA2 activity help ensure appropriate diagnosis. To date, more than 60 mutations of the ADA2 gene have been identified in the literature [12], and the majority of DADA2 patients have compound heterozygous missense mutations. The most common variants of the disease are p.Gly47Ala (p.G47A), p.Gly47Arg (p.G47R), p.Tyr453Cys (p.Y453C), and p.Arg169Gln (p.R169Q) [2,13]. The genetic testing of our patient revealed two pathogenic variants, c.882-2A>G splice acceptor (homozygous). In some cases, deletion mutation may lead to the deletion of exon 7, a mutational hotspot, which may reduce transcription and produce severe functional damage [10,13].

In almost all patients who received treatment with biologic TNF inhibitors, fever episodes, vasculopathy, and stroke prevention were successfully managed [2]. Less clear evidence has been recorded with the cure or reversal of immunodeficiency and cytopenia [11] and opportunistic infections [11,14]. Patients who present with hematological illness and immunodeficiency and who do not respond to TNF inhibitors may have the option to receive a hematopoietic stem cell transplant. Gene therapy or recombinant ADA2 protein may be used in future therapeutic strategies. More research is required on the role of ADA2 and the pathophysiology of ADA2 deficiency to create targeted therapeutics. ADA2 may serve as a novel target for antiangiogenic therapy in some types of human cancer, which could ultimately provide individuals suffering from this terrible affliction with the best possible care [15].

## Conclusions

In conclusion, we present the first case in Saudi Arabia of an adult patient diagnosed with DADA2 with a unique gene mutation. Patients with adult-onset DADA2 usually have a vague presentation and a relatively narrower phenotype range of symptoms which produce additional challenges for the physician to add DADA2 to the list of differentials. Timely gene sequencing and measurements of ADA2 activity might assist in accurate diagnosis. We suggest further studies investigate the genotype-phenotype association, possible clinical presentation, and the development of curative treatments for those cases.

## References

[1] Zhou Q, Yang D, Ombrello AK (2014). Early-onset stroke and vasculopathy associated with mutations in ADA2. N Engl J Med.

[2] Navon Elkan P, Pierce SB, Segel R (2014). Mutant adenosine deaminase 2 in a polyarteritis nodosa vasculopathy. N Engl J Med.

[3] Lee PY (2018). Vasculopathy, immunodeficiency, and bone marrow failure: the intriguing syndrome caused by deficiency of adenosine deaminase 2. Front Pediatr.

[4] Meyts I, Aksentijevich I (2018). Deficiency of adenosine deaminase 2 (DADA2): updates on the phenotype, genetics, pathogenesis, and treatment. J Clin Immunol.

[5] Rama M, Duflos C, Melki I (2018). A decision tree for the genetic diagnosis of deficiency of adenosine deaminase 2 (DADA2): a French reference centres experience. Eur J Hum Genet.

[6] Kendall JL, Springer JM (2020). The many faces of a monogenic autoinflammatory disease: adenosine deaminase 2 deficiency. Curr Rheumatol Rep.

[7] Aksentijevich I, Sampaio Moura N, Barron K (2019). Adenosine Deaminase 2 Deficiency. https://www.ncbi.nlm.nih.gov/books/NBK544951/.

[8] Zhang B, Sun Y, Xu N (2021). Adult-onset deficiency of adenosine deaminase 2 - a case report and literature review. Clin Rheumatol.

[9] Sharma A, Naidu G, Sharma V (2021). Deficiency of adenosine deaminase 2 in adults and children: experience from India. Arthritis Rheumatol.

[10] van Well GTJ, Kant B, van Nistelrooij A (2019). Phenotypic variability including Behçet's disease-like manifestations in DADA2 patients due to a homozygous c.973-2A>G splice site mutation. Clin Exp Rheumatol.

[11] Hashem H, Kumar AR, Müller I (2017). Hematopoietic stem cell transplantation rescues the hematological, immunological, and vascular phenotype in DADA2. Blood.

[12] (2018). ADA2 gene: adenosine deaminase 2. https://medlineplus.gov/genetics/condition/adenosine-deaminase-2-deficiency/.

[13] Trotta L, Martelius T, Siitonen T (2018). ADA2 deficiency: clonal lymphoproliferation in a subset of patients. J Allergy Clin Immunol.

[14] Cipe FE, Aydogmus C, Serwas NK, Keskindemirci G, Boztuğ K (2018). Novel mutation in CECR1 leads to deficiency of ADA2 with associated neutropenia. J Clin Immunol.

[15] Zhu C, Chrifi I, Mustafa D (2017). CECR1-mediated cross talk between macrophages and vascular mural cells promotes neovascularization in malignant glioma. Oncogene.

